# Communicating about paediatric infectious diseases at the beginning of the 20th century

**DOI:** 10.3892/mi.2025.235

**Published:** 2025-04-16

**Authors:** Ioannis N. Mammas, Simon B. Drysdale, Maria Theodoridou, Demetrios A. Spandidos

**Affiliations:** 1 First Department of Paediatrics, University of Athens School of Medicine, 11527 Athens, Greece; 2 Laboratory of Clinical Virology, Medical School, University of Crete, 71003 Heraklion, Greece; 3 Paediatric Clinic, Aliveri, 34500 Island of Euboea, Greece; 4 Oxford Vaccine Group, Department of Paediatrics, University of Oxford, Oxford, OX3 9DU, UK; 5 NIHR Oxford Biomedical Research Centre, Oxford, OX3 9DU, UK

**Keywords:** Charta of paediatric infectious diseases, prevention, hygiene, 20th century, Institute of Paediatric Virology

## Abstract

The Charta of paediatric infectious diseases, which was printed in Athens, Greece in 1912, contains instructions for school students on the prevention of paediatric infectious diseases occurring in Greece at the beginning of the 20th century. It consists of four sections: i) The official circular of the Department of the School of Hygiene of the Hellenic Ministry of Education on the protection of school students from acute infectious diseases signed by the minister on January 31, 1912; ii) an introductory section on the definition of infectious diseases, the modes of their transmission and the conditions required for the inactivation of microbial activity; iii) a section with general preventative measures against paediatric infectious diseases; and iv) a section with information and specialized measures against specific paediatric infectious diseases. It also contains colourful images of children with different infectious diseases, including smallpox, measles, diphtheria, varicella, eye trachoma and other infectious diseases involving the skin. The Charta of paediatric infectious diseases is exhibited at the Museum of School Students' Life in Aliveri, on the island of Euboea in Greece and was recently reprinted in the context of the 10th workshop on paediatric virology organized by the Institute of Paediatric Virology on November 9, 2024.

## Charta of paediatric infectious diseases

Among the exhibited collection of the Museum of School Students' Life, which is located in Aliveri, on the island of Euboea in Greece, is the Charta of paediatric infectious diseases printed in Athens, Greece in 1912. The map (length, 95 cm; height, 65 cm), which is entitled ‘Instructions for the protection of school students from infectious diseases’ consists of four sections: i) The official circular of the Hellenic Ministry of Education on the protection of school students from acute infectious diseases, which was designed by the Department of School Hygiene and signed by Minister Apostolos G. Alexandris on January 31, 1912; ii) an introductory section on the definition of infectious diseases, the modes of their transmission and the conditions required for the inactivation of microbial activity; iii) a section with general preventative measures against paediatric infectious diseases; and iv) a section with information and specialized measures against specific paediatric infectious diseases. The map also contains 11 colourful images of children with different infectious diseases, including smallpox, measles, diphtheria, varicella, trachoma and other infections involving the skin. It was written in the Greek language using the polytonic system; neither its creator nor editor are mentioned.

The Charta of paediatric infectious diseases dates back to the beginning of the last century, when the discovery of microbes and the principles of hygiene were considered as the most significant achievements of medicine against paediatric infectious diseases (1,2). From the official circular of the Hellenic Ministry of Education, which has been included in the Charta, one can read: ‘Until a few years ago, science, which was ignorant of the causes and modes of transmission of infectious diseases, was completely unable to prevent them. Since then, when the pathogenic agents of these diseases were discovered, i.e., microbes, infectious diseases ceased to be fatal events, which one inevitably had to suffer and accept. Most of the infectious diseases, if not all, can be avoided with the necessary care for their prevention and the means taught by the science of hygiene.’

During the previous years, in Europe, as well as in the USA, the value of hygiene in public areas, including schools had already achieved an increasing interest and hygiene teaching had been included into the general educational programmes of several countries coordinated by specialized school health services (3). Primary and secondary schools thus emerged as a natural arena for innovative, at that time, health educational efforts and by associating the concept of hygiene principles into life-practice. The map describes the first preventative efforts in Greece against paediatric infections, performed by the newly established, at that time, Department of School Hygiene of the Hellenic Ministry of Education (3). During the following years, this service was further organized and developed, implementing specific laws, orders, circulars, instructions, reports and notes, as they are well described in the Code of School Hygiene written in 1922 by Dr Emmanuel N. Lampadarios, Director of the Department of School Hygiene of the Hellenic Ministry of Education in Greece, at that time (4).

The Charta of paediatric infectious diseases focuses on the value of appropriate ventilation, lighting and cleanliness in schools, the students' compliance with the hygiene principles, the isolation of students with infectious diseases and the closure of schools as a preventative measure against paediatric infectious diseases >100 years before the current coronavirus disease 2019 (COVID-19) pandemic (5). It uses medical terms, such as ‘prevention’, ‘immunity’ and ‘immunization’, which are still in use in medical practice today; however, at that time, they were not completely understood or widely developed. The description of different infectious diseases, such as measles, varicella, smallpox and diphtheria, their mode of transmission and instructions for their preventative are presented in detail. The infectious diseases included in the Charta were the most common causes of paediatric mortality and morbidity (1,2). In 1912, although pathogenic bacteria had been identified, antibiotics for the management of bacterial pathogens had not been discovered. Moreover, systematic vaccination against infections in childhood had not been introduced (6-8).

Notably, the Charta of paediatric infectious diseases sought the discovery of the causative agents of viral infections; at that time, the science of virology was at an embryonic stage (6-8). Viruses, such as influenza viruses, were not able to be identified. According to the map, ‘until now the causative agent of measles has not been discovered yet, however it seems that it is located in sputum, in the mucus of the nose and pharynx and in the scales of the skin and is transmitted by direct and indirect contact with patients’. Regarding varicella, ‘its microbe remains unknown’, while as far as smallpox is concerned, ‘until now its specific microbe has not been revealed yet’.

**Figure 1:**
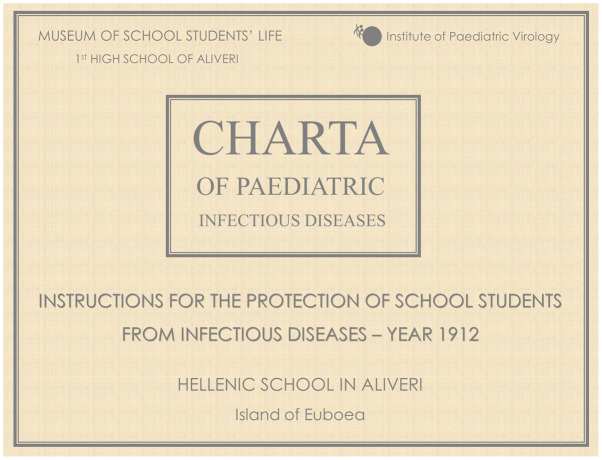
The Charta of paediatric infectious diseases (1912) reprinted by the Museum of School Students' Life in Aliveri on the island of Euboea in Greece in collaboration with the Institute of Paediatric Virology (IPV, http://www.paediatricvirology.org) in the context of the 10th workshop on paediatric virology held on November 9, 2024.

In the context of the recent 10th workshop on paediatric virology, the Charta of paediatric infectious diseases (Fig. 1) was reprinted by the Museum of School Students' Life in collaboration with the Institute of Paediatric Virology (9). The reprinting of the map also involved the searching of the Hellenic medical literature from the beginning of the 20th century on paediatric infectious diseases and all laws and official circulars by the Hellenic Ministry of Education of that period on infection prevention and hygiene (4). At that time in Greece, paediatrics had not yet been recognized as an official specialty of medicine; however, the map contains a very precise level of information on the value of infection prevention and hygiene in schools highlighting their significant role in the management of paediatric infections occurring in Greece at the beginning of the 20th century. The Charta of paediatric infectious diseases is indeed an excellent way of communicating science to children, teachers and other school personnel.

## Data Availability

Not applicable.
